# Differences in Peripheral Blood Gene Expression of Xinjiang Brown Cattle with Varying Somatic Cell Counts

**DOI:** 10.3390/biology15110830

**Published:** 2026-05-25

**Authors:** Mengjie Yan, Dan Wang, Shengchao Ma, Jiangkun Wang, Lei Xu, Menghua Zhang, Xixia Huang

**Affiliations:** College of Animal Science, Xinjiang Agricultural University, Urumqi 830052, China

**Keywords:** somatic cell counts (SCCs), mastitis, Oxford Nanopore Technologies (ONT), *CXCL2*, Xinjiang Brown cattle

## Abstract

Bovine mastitis remains a major impediment to optimal dairy production. Somatic cell count (SCC) is commonly used as an indicator of mammary gland inflammation, while milk microbiota may also reflect mastitis-related changes. Here, we employed Oxford Nanopore full-length transcript sequencing to delineate the peripheral blood transcriptomic landscape of Xinjiang Brown cattle stratified by high (SCC ≥ 1,000,000 cells mL^−1^) and low (SCC ≤ 200,000 cells mL^−1^) SCCs, with the objective of identifying candidate genes underpinning mastitis resistance. We identified 226 differentially expressed genes and 441 differentially expressed transcripts. Genes in the high-SCC group were prominently enriched in immune response pathways and chemokine signalling cascades. Protein–protein interaction network analysis further delineated a core module of ten immune-related genes, including *CCL4*, *IL1B* and *CXCL2*. Integrative analysis with complementary second-generation sequencing data pinpointed *CXCL2* as a high-priority candidate. Subsequent RT–qPCR and enzyme-linked immunosorbent assay (ELISA) validation revealed that *CXCL2* expression was significantly elevated both in high-SCC individuals and in an LPS-induced bovine mammary epithelial cell inflammation model. Collectively, these findings establish *CXCL2* as a putative molecular marker for mastitis resistance breeding and provide a foundational resource for deciphering the molecular mechanisms governing mammary health.

## Simple Summary

Mastitis is a common and costly disease in dairy cows, and somatic cell count (SCC) is widely used to assess udder health. In this study, we used Oxford Nanopore full-length transcript sequencing to compare the blood gene expression profiles of Xinjiang Brown cows with high- and low-SCCs. We identified hundreds of genes and transcripts that differ between these groups, with high-SCC cows showing increased activity in immune-related pathways. Among these, *CXCL2* emerged as a key candidate gene. Laboratory experiments confirmed that *CXCL2* levels are higher in cows with high-SCCs and in inflamed mammary cells. These results suggest that *CXCL2* could serve as a useful marker for selecting cows resistant to mastitis and help us better understand the biology of udder health.

## 1. Introduction

Bovine mastitis remains one of the most prevalent and economically damaging diseases affecting dairy herds worldwide. The condition exacts a heavy toll on production efficiency, primarily through reduced milk yield, impaired milk quality, extended calving intervals, compromised fertility, and shortened productive lifespans. It has been estimated that daily milk yield declines by 1.0–2.5 kg as early as two weeks before clinical onset, culminating in total lactation losses of 110–552 kg—deficits that are both substantial and irreversible [1]. Somatic cell counts (SCCs) show a strong genetic correlation with mastitis incidence, with reported estimates ranging from 0.59 to 0.85 [2]. Consequently, SCC is widely adopted not only as a reliable proxy for udder health and mastitis risk, but also as a key selection trait in milk quality monitoring and genetic improvement programmes [3,4]. In practice, SCC < 500,000 cells mL^−1^ is generally considered indicative of a healthy mammary gland, SCC > 1,000,000 cells mL^−1^ signals clinical mastitis, and intermediate values denote subclinical intramammary infection [5]. The intramammary ecosystem harbours a diverse microbial community that, under physiological conditions, maintains a balanced state; this microbiota—particularly the commensal populations—contributes to the maintenance of local immune homeostasis [6]. Disruption of this equilibrium by invading pathogens triggers an inflammatory response, which is accompanied by a marked influx of somatic cells into mammary tissue. The resulting elevation in SCC therefore reflects the convergence of local mammary immunity and systemic immune status, representing a key host defence mechanism against microbial challenge. Understanding how mammary immune regulation differs across SCC strata at the molecular level is of fundamental importance for improving udder health and enhancing production performance. Although mammary tissue represents an ideal material for studying udder health traits, its large-scale acquisition under practical production conditions remains challenging. In contrast, blood samples are readily accessible and enable reproducible analyses, with their transcriptomic information partially reflecting the systemic metabolic, immune, and endocrine status. Moreover, circulating blood leukocytes play a critical role in the onset, progression, and resolution of bovine mastitis [7], offering a feasible avenue for investigating the regulatory mechanisms underlying milk composition and health traits.

The Xinjiang Brown cow is a Chinese dual-purpose breed valued for its adaptation to cold climates, tolerance of coarse feedstuffs, and high-quality meat and milk. Our previous investigations in this population have identified several candidate genes associated with mastitis resistance. For instance, Zhou et al. [8] employed a genome-wide association study (GWAS) to detect three single-nucleotide polymorphisms (SNPs) significantly associated with somatic cell score (SCS) in Xinjiang Brown cattle, located in the vicinity of *LOC104969301*, *FHIT* and *DYRK2*. Subsequent pyrosequencing analysis revealed that differential methylation of the *FHIT* promoter modulates gene expression, thereby influencing mastitis susceptibility; specifically, decreased methylation levels in the *FHIT* promoter region were associated with enhanced resistance to mastitis in this breed [9]. In parallel, Wang et al. [10] proposed that *TRAPPC9* and *CD4* are negatively regulated in relation to mastitis occurrence and identified these genes as two novel DNA methylation targets implicated in mastitis susceptibility in Xinjiang Brown cattle. Collectively, these findings have contributed candidate molecular markers for genetic improvement programmes aimed at enhancing mastitis resistance in this breed.

**Figure 1:**
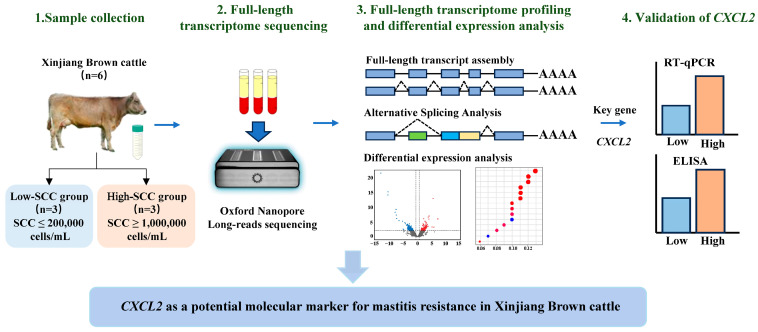
Schematic flowchart of the experimental design and workflow of this study.

RNA sequencing (RNA-Seq) based on high-throughput platforms has been widely applied to investigate economically important traits in livestock and poultry. However, second-generation transcriptome sequencing technologies are not always entirely accurate during transcript assembly, and their short read lengths typically preclude the recovery of full-length transcripts. To overcome these limitations, full-length transcriptome sequencing based on single-molecule real-time sequencing technologies has emerged [11]. Compared with second-generation approaches, this technology offers longer reads and higher throughput; it eliminates the need for RNA fragmentation during library preparation and circumvents transcript assembly during data analysis, thereby enabling the capture of complete, full-length transcript isoforms [12]. These features confer substantial advantages in resolving transcript diversity, identifying novel transcripts, and deciphering the architecture of complex immune-related genes. In the present study, we employed Oxford Nanopore Technology (ONT) long-read transcriptome sequencing to systematically compare gene expression profiles in peripheral blood of Xinjiang Brown cattle stratified by SCCs. Our objective was to identify key candidate genes associated with differential SCC levels, thereby providing a theoretical foundation and data resource for elucidating the molecular mechanisms underlying mammary health traits and for advancing health-oriented breeding strategies in Xinjiang Brown cattle (Figure 1).

## 2. Materials and Methods

### 2.1. Sample Collection

All Xinjiang Brown cattle used in this study were obtained from the Xinjiang Yili Brown Cattle Breeding Farm in Xinjiang, China. The selected individuals were lactating multiparous cows in mid-to-late lactation and were maintained under uniform feeding and management conditions. Blood samples were collected using 10 mL EDTA tubes, and milk samples were collected using 50 mL centrifuge tubes. Following collection, blood samples were centrifuged at 3500 rpm for 15 min. The buffy coat was immediately separated into new tubes after adding 1 mL Trizol (Thermo Fisher, Waltham, MA, USA). The samples were then promptly transported on dry ice to the laboratory and stored at −80 °C for subsequent RNA extraction. Milk samples were collected from all four mammary quarters, thoroughly mixed, and then subjected to compositional analysis and SCC determination. Based on SCC values, individuals were classified into two groups: low-SCC (≤200,000 cells mL^−1^) and high-SCC (≥1,000,000 cells mL^−1^). A total of six Xinjiang Brown cattle in mid-to-late lactation were selected, with three animals per group.

### 2.2. Library Construction and Sequencing

Total RNA was extracted from peripheral blood leukocytes and assessed for quality prior to library preparation. Full-length cDNA was synthesized using a high-fidelity reverse transcriptase, followed by 14 cycles of PCR amplification with LongAmp Taq (NEB, Ipswich, MA, USA) and barcoded primers containing specific adapters (cDNA-PCR Sequencing Kit, SQK-LSK110 and EXP-PCB096; Oxford Nanopore Technologies, Oxford, UK). The resulting amplicons were ligated to Oxford Nanopore sequencing adapters using T4 DNA ligase (NEB) and subsequently purified with Agencourt XP beads (Beckman Coulter, Brea, CA, USA). The final purified library was loaded onto FLO-MIN109 flow cells and sequenced on the PromethION platform (Oxford Nanopore Technologies).

### 2.3. Data Quality Control and Read Mapping

Raw sequencing data generated by ONT were subjected to quality control procedures. Reads with an average quality score below 6 or a length shorter than 350 bp were discarded. Ribosomal RNA (rRNA) reads were identified and removed by aligning all reads against an rRNA database. Following initial quality filtering, full-length non-chimeric (FLNC) transcripts were identified based on the presence of adapter sequences at both ends of each read. Reads containing both the 5′ primer (TTTCTGTTGGTGCTGATATTGC) and the 3′ primer (GAAGATAGAGCGACAGGCAAGT) at their termini were classified as full-length transcripts. During full-length transcript sequencing, the stable polyA structure at the 3′ end ensures relatively high integrity of this region; however, degradation may occur at the 5′ end, leading to variations in 5′ termini among different copies of the same transcript. This heterogeneity can result in the assignment of these copies to distinct clusters rather than a single consensus sequence, thereby generating redundancy in the transcript dataset. To address this redundancy, all FLNC reads were aligned to the bovine reference genome (*Bos taurus*, GCF_002263795.3_ARS_UCD2.0) using minimap2 (version 2.16) [13]. The resulting alignments were processed to remove redundant sequences using the cDNA_Cupcake package (version 29.0.0). Sequences with identity <0.9 or coverage <0.85 were filtered out to ensure the accuracy and reliability of downstream analyses.

### 2.4. Identification of Alternative Splicing

Alternative splicing (AS) events were identified using Astalavista software (version 3.2-0) [14]. The analysis encompassed five major types of splicing events: alternative 3′ splice sites (A3SSs), alternative 5′ splice sites (A5SSs), exon skipping (SE), intron retention (RI) and mutually exclusive exons (MXEs). Differentially AS events were defined by a threshold of |ΔPSI| > 0.1 and *p* < 0.01, where PSI (percent spliced in) represents the inclusion level of a given exon or splice junction. Genes harbouring such differential splicing events were designated as differentially spliced genes (DSGs).

### 2.5. Differential Expression Analysis of Genes and Transcripts

Full-length reads were quantified by alignment to the bovine reference genome. Following alignment, read counts for genes and transcripts were obtained and subsequently normalized for downstream visualization. Differential expression analysis was performed using the negative binomial regression algorithm implemented in the DESeq2 package (version 1.40.2). Genes and transcripts with a *p* < 0.01 and a fold change ≥1.5 were considered significantly differentially expressed.

### 2.6. Functional Annotation and Enrichment Analysis of DEGs and DETs

Functional annotation and pathway enrichment analysis were performed on the sets of differentially expressed genes (DEGs) and differentially expressed transcripts (DETs) identified from the comparison of high- versus low-SCC groups in Xinjiang Brown cattle. Gene Ontology (GO) annotation and Kyoto Encyclopedia of Genes and Genomes (KEGG) pathway enrichment analyses were conducted using the DAVID Bioinformatics Resources (https://davidbioinformatics.nih.gov/tools.jsp, accessed on 16 March 2026). To investigate protein–protein interaction (PPI) networks, the DEGs were mapped to the STRING database (https://cn.string-db.org/, accessed on 16 March 2026) using the BLASTx (version 2.15.0) algorithm to identify interactions with relevant orthologous species. The resulting interaction data were then imported into Cytoscape (version 3.9.1) for visualization and network analysis. Hub genes within the network were identified based on the Degree algorithm, enabling the prioritization of key nodes and providing insights into the complex relationships and potential biological roles of the candidate genes.

### 2.7. Cell Culture

Mac-T cells used in this study were obtained from Qingqi Biotechnology Development Co., Ltd. (Shanghai, China). For revival, cryovials containing the cells were retrieved from liquid-nitrogen storage and immediately immersed in a 37 °C water bath with gentle agitation until completely thawed. All subsequent steps were performed in a laminar flow hood. The thawed cell suspension was transferred to a centrifuge tube, diluted with at least five volumes of culture medium and mixed thoroughly. Cells were pelleted by centrifugation at 1000 rpm for 10 min, after which the supernatant was discarded. The cell pellet was resuspended in fresh culture medium and seeded into culture dishes at a split ratio of 1:10–1:15, achieving a final cell density of approximately 1–5 × 10^5^ cells mL^−1^. Cells were maintained at 37 °C in a humidified incubator with 5% CO_2_.

### 2.8. Establishment of an In Vitro Cellular Inflammation Model

Cellular inflammation was induced following an established protocol developed by our research group. Briefly, phosphate-buffered saline (PBS) was sterilized and used to dilute lipopolysaccharide (LPS) to a final concentration of 10 ng/μL. The LPS working solution was then applied to Mac-T cells cultured in 12-well plates at 70–80% confluence. Cells were incubated with LPS for 3 h at 37 °C in a humidified atmosphere containing 5% CO_2_ prior to subsequent analyses.

### 2.9. RNA Extraction from Cultured Cells

Following LPS treatment for 3 h, the culture medium was removed and cells were washed three times with PBS. Subsequently, 1 mL of Trizol reagent was added to each well, and cells were lysed by repeated pipetting until the cell lysate appeared homogeneous. The resulting lysate was transferred to a 2 mL centrifuge tube. Total RNA was then extracted according to the manufacturer’s instructions.

### 2.10. RT-qPCR

To investigate the inflammatory expression pattern of *CXCL2*, its transcript levels were examined both in peripheral blood of Xinjiang Brown cattle with high- and low-SCC and in an LPS-induced cellular inflammation model using RT-qPCR (Table 1). Total RNA was reverse transcribed into cDNA using the PrimeScript™ RT Reagent Kit with gDNA Eraser (Perfect Real Time, TaKaRa, Kusatsu, Japan) according to the manufacturer’s instructions. Quantitative PCR was subsequently performed using the TB Green^®^ Premix Ex Taq™ II (Tli RNaseH Plus) kit (TaKaRa) in a 25 μL reaction volume. All reactions were carried out in accordance with the manufacturer’s protocols.

### 2.11. ELISA for CXCL2 Protein Quantification

To quantify CXCL2 protein levels, cell culture supernatants were collected from Mac-T cells following 3 h of LPS treatment. The supernatants were centrifuged at 3000 rpm for 20 min at 4 °C to remove cellular debris and particulate matter. The clarified supernatants were then subjected to enzyme-linked immunosorbent assay (ELISA) using a Bovine CXC Chemokine Ligand 2 (CXCL2) ELISA kit (Shanghai Enlink Biotechnology Co., Ltd., Shanghai, China), following the manufacturer’s instructions. CXCL2 concentrations were determined based on a standard curve generated from provided standards.

## 3. Results

### 3.1. Transcriptome Sequencing Data Quality and Mapping Statistics

**Figure 2:**
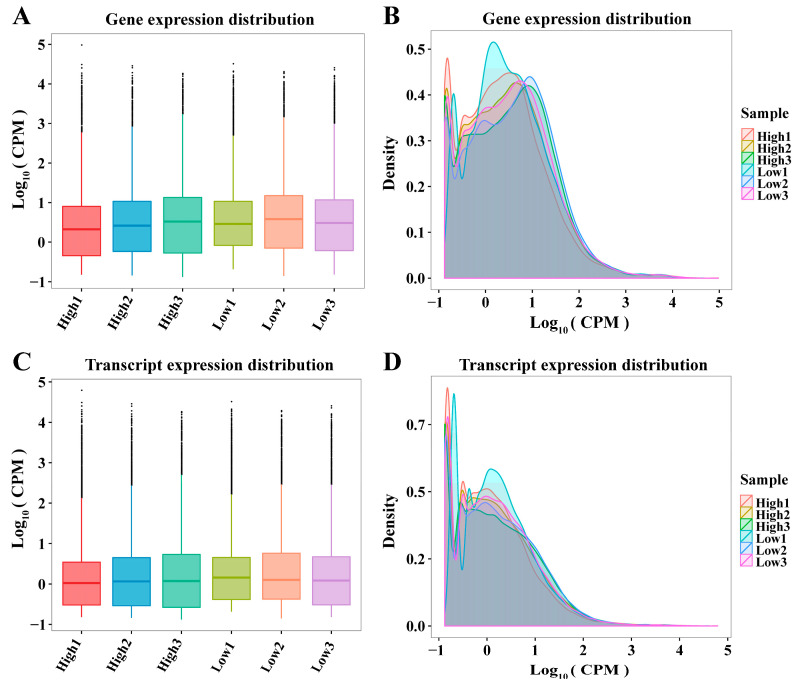
Distribution of gene and transcript expression levels across samples. (**A**) Box plot showing the distribution of gene expression levels across the six samples; (**B**) density curve illustrating the distribution of gene expression levels; (**C**) box plot depicting transcript expression levels across samples; (**D**) density curve showing the distribution of transcript expression levels.

A total of six Xinjiang Brown cattle were included in this study, classified into two groups based on SCC, high-SCC (high 1–3; SCC ≥ 1,000,000 cells mL^−1^) and low-SCC (low 1–3; SCC ≤ 200,000 cells mL^−1^), with three biological replicates per group. RNA samples isolated from peripheral blood leukocytes of all individuals met the quality criteria for concentration, purity and integrity (Table S1), and were subsequently used for library construction and sequencing. After filtering raw fastq data to remove short fragments, low-quality reads and ribosomal RNA sequences, a total of 5,475,643 to 8,447,986 clean reads were obtained per sample, with each sample yielding at least 5.96 GB of clean data. The length distribution of clean reads showed that fragments of 1 kb were the most abundant, with an overall mean read length of 786 bp, followed by fragments of 2 kb and 3 kb. The maximum read length reached 190,233 bp, with an average maximum length of 136,472 bp across samples. The mean N50 length was 0.8 kb for all groups (Table S2, Figure S1). Full-length reads were identified based on the presence of adapter sequences at both ends of each read. Between 5,475,643 and 8,447,986 full-length reads were obtained per sample, with full-length ratios exceeding 92% in all cases (Table S3). Clean reads were aligned to the bovine reference genome, yielding mapping rates greater than 95% for each sample and an average mapping rate of 97.3% (Table S4), indicating high coverage and reliability of the sequencing data. Collectively, these results demonstrate that the sequencing depth and quality were sufficient for subsequent analyses. To assess global gene and transcript expression patterns across samples, box plots and density curves were generated based on normalized expression values. No significant differences were observed in the interquartile ranges or medians among the six samples, and the areas under the density curves were similarly distributed across groups (Figure 2). These findings indicate that overall gene and transcript expression levels were comparable between the high- and low-SCC groups, with no systemic bias introduced by sequencing or sample processing.

**Figure 3:**
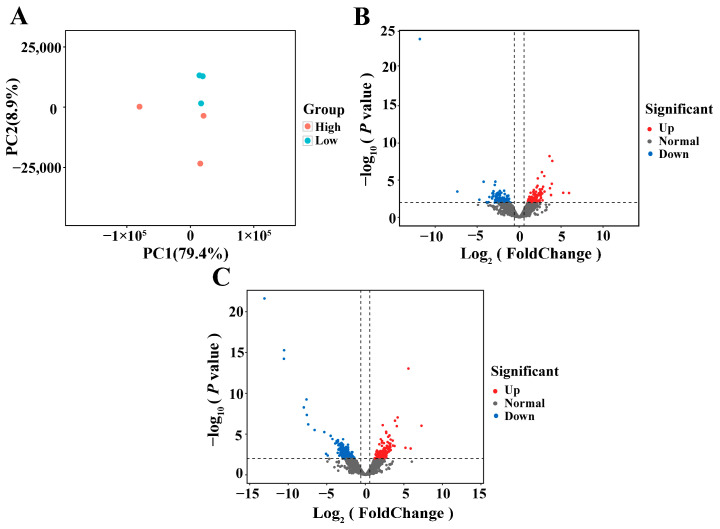
Transcriptional landscape and differential expression analysis between high- and low-SCC groups. (**A**) PCA of the six samples based on global gene expression profiles; (**B**) volcano plot displaying DEGs between high- and low-SCC groups; (**C**) volcano plot showing DETs.

PCA revealed that the three samples from the low-SCC group clustered tightly together, whereas those from the high-SCC group exhibited greater dispersion. Notably, projection of all six samples onto the second principal component (PC2) axis resulted in clear separation into two distinct clusters corresponding to the high- and low-SCC groups (Figure 3A). Comparative transcriptome analysis between the high- and low-SCC groups identified a total of 226 DEGs (*p* < 0.01, fold change ≥ 1.5), of which 111 were upregulated and 115 were downregulated in the high-SCC group relative to the low-SCC group (Figure 3B). In addition, 441 DETs were identified, comprising 135 upregulated and 306 downregulated transcripts in the high-SCC group (Figure 3C).

### 3.2. Differential Expression Gene Clustering and Protein–Protein Interaction Network Analysis

**Figure 4:**
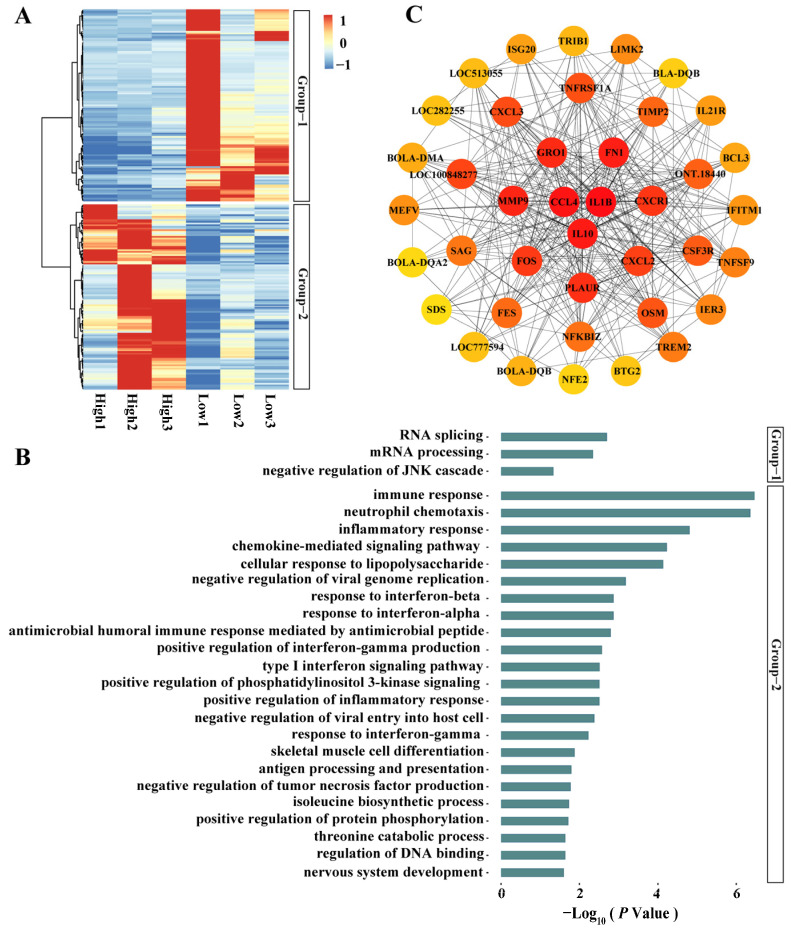
Clustering, functional enrichment and protein–protein interaction network of DEGs. (**A**) Heatmap showing hierarchical clustering of the 226 DEGs across six samples; (**B**) GO enrichment analysis of DEGs in Group-1 and Group-2; (**C**) PPI network of DEGs constructed using the STRING database and visualized with Cytoscape.

Hierarchical clustering of the 226 DEGs revealed two distinct expression modules, designated Group-1 and Group-2 (Figure 4A). Group-1 comprised genes with elevated expression in the low-SCC group, whereas Group-2 consisted of genes upregulated in the high-SCC group. Functional annotation indicated that Group-1 genes were primarily associated with biological processes such as RNA splicing, mRNA processing and negative regulation of the JNK cascade. In contrast, Group-2 genes were significantly enriched in immune-related pathways and processes, including immune response, neutrophil chemotaxis, inflammatory response, and chemokine-mediated signalling pathways (Figure 4B). PPI network analysis identified ten hub genes: *CCL4*, *IL1B*, *IL10*, *GRO1*, *FN1*, *CXCR1*, *CXCL2*, *PLAUR*, *FOS* and *MMP9* (Figure 4C). The majority of these hub genes are known to participate in immune and inflammatory responses, further supporting the functional enrichment observed in Group-2. Collectively, these results demonstrate that DEGs upregulated in the high-SCC group are predominantly involved in immune response, inflammatory response and chemokine activity. The identification of immune-related hub genes through PPI analysis provides additional validation of this finding and highlights potential key regulators of mastitis susceptibility in Xinjiang Brown cattle.

### 3.3. Functional Enrichment Analysis of Differentially Expressed Genes

To elucidate the biological functions and pathways associated with mastitis susceptibility, GO and KEGG enrichment analyses were performed on the 226 DEGs identified between high- and low-SCC groups.

**Figure 5:**
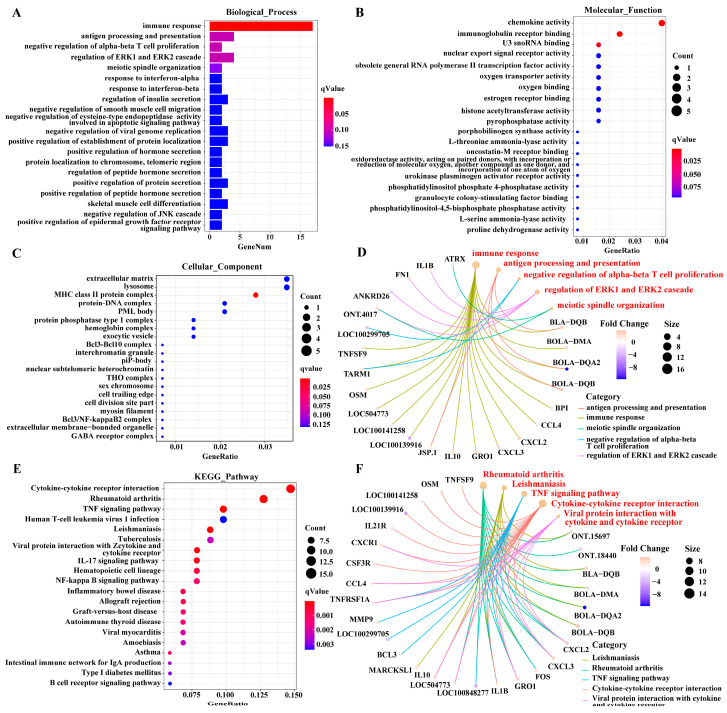
GO and KEGG pathway enrichment analysis of DEGs. (**A**) Bar plot showing the top 20 enriched GO terms in the BP category for DEGs between high- and low-SCC groups; (**B**) bar plot depicting the top 20 enriched GO terms in the MF category; (**C**) bar plot illustrating the top 20 enriched GO terms in the CC category; (**D**) cnetplot visualizing the relationships between the top five enriched BP terms and their associated DEGs; (**E**) dot plot displaying KEGG pathway enrichment analysis results for DEGs; (**F**) cnetplot illustrating the connections between the top five enriched KEGG pathways and their associated DEGs.

GO enrichment analysis revealed that DEGs were significantly enriched in several biological process (BP) terms, including immune response, antigen processing and presentation, negative regulation of alpha-beta T-cell proliferation, and regulation of the ERK1 and ERK2 cascade (Figure 5A). In the molecular function (MF) category, enriched terms included chemokine activity, immunoglobulin receptor binding, U3 snoRNA binding and nuclear export signal receptor activity (Figure 5B). For cellular component (CC), DEGs were primarily localized to the extracellular matrix, lysosome, MHC class II protein complex, and protein–DNA complex (Figure 5C). The five most significantly enriched BP terms were associated with key immune-related genes, including *CXCL2*, *CXCL3*, *GRO1*, *IL10*, *BPI*, *TARM1* and *IL1B* (Figure 5D). KEGG pathway enrichment analysis demonstrated that DEGs were predominantly enriched in cytokine–cytokine receptor interaction, rheumatoid arthritis, TNF signalling pathway, human T-cell leukaemia virus 1 infection and leishmaniasis (Figure 5E). The top five enriched pathways featured genes such as *CXCL2*, *CXCL3*, *FOS*, *GRO1*, *IL1B*, *IL10*, *CXCR1* and *IL21R* (Figure 5F).

Collectively, these results indicate that DEGs upregulated in the high-SCC group are primarily involved in immune response and antigen–antibody reaction pathways, with core genes including *CXCL2*, *CXCL3*, *GRO1*, *IL1B* and *IL10*.

### 3.4. Differential Expression Transcript Clustering and Protein–Protein Interaction Network Analysis

**Figure 6:**
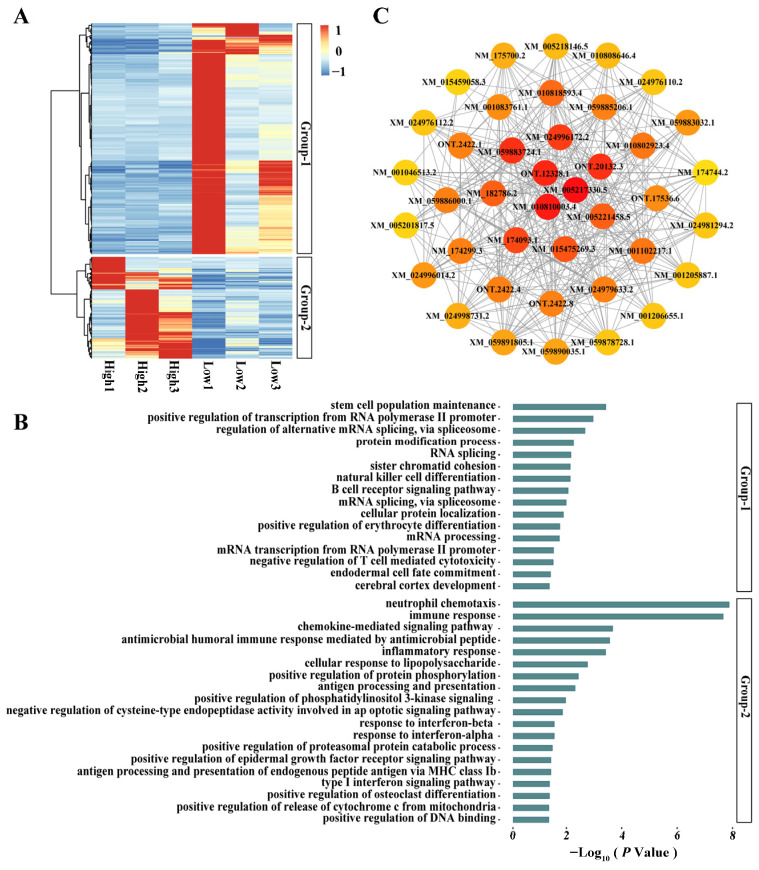
Clustering, functional enrichment and protein–protein interaction network of DETs. (**A**) Heatmap showing hierarchical clustering of the 441 DETs across six samples; (**B**) GO enrichment analysis of genes corresponding to DETs in Group-1 and Group-2; (**C**) PPI network of proteins encoded by DETs, constructed using the STRING database and visualized with Cytoscape.

Hierarchical clustering of the 441 DETs revealed two distinct expression modules, designated Group-1 and Group-2 (Figure 6A). Group-1 comprised transcripts with elevated expression in the low-SCC group, whereas Group-2 consisted of transcripts upregulated in the high-SCC group. GO enrichment analysis of the genes corresponding to these transcript modules revealed distinct functional landscapes. Group-1-associated genes were primarily enriched in biological processes related to stem cell population maintenance, positive regulation of transcription from RNA polymerase II promoter, regulation of alternative mRNA splicing via the spliceosome, the protein modification process, and RNA splicing (Figure 6B). In contrast, Group-2-associated genes were significantly enriched in immune-related processes, including neutrophil chemotaxis, immune response, chemokine-mediated signalling pathway, antimicrobial humoral immune response mediated by antimicrobial peptide and inflammatory response (Figure 6B).

PPI network analysis of all DET-encoding genes identified ten hub transcripts: ONT.12328.1, XM_010810003.4, XM_005217330.5, ONT.20132.3, XM_024996172.2, XM_059883724.1, NM_182786.2, NM_174093.1, XM_015475269.3 and XM_005221458.5 (Figure 6C). These transcripts correspond to the following genes: *IL10*, *RANBP2*, *PTPRC*, *ATRX*, *SMC5*, *FOS*, *IL1B*, *TPR* and *ERBIN*.

### 3.5. GO and KEGG Enrichment Analysis of Differentially Expressed Transcripts

To further characterize the functional landscape of transcriptomic alterations associated with SCCs, GO and KEGG enrichment analyses were performed on the genes corresponding to the 441 DETs identified between high- and low-SCC groups.

**Figure 7:**
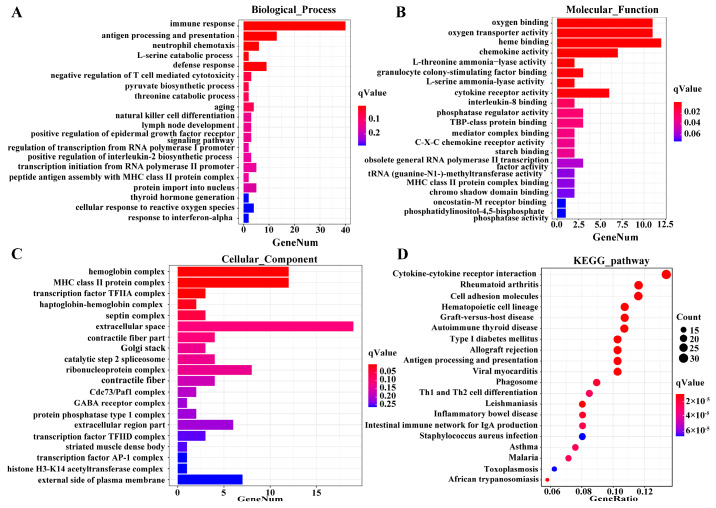
GO and KEGG pathway enrichment analysis of DETs. (**A**) Bar plot showing the top 20 enriched GO terms in the BP category for genes corresponding to DETs between high- and low-SCC groups; (**B**) bar plot depicting the top 20 enriched GO terms in the MF category; (**C**) bar plot illustrating the top 20 enriched GO terms in the CC category; (**D**) dot plot displaying the top 20 enriched KEGG pathways for DET-associated genes.

GO enrichment analysis revealed that DET-associated genes were significantly enriched in several biological process (BP) terms, including immune response, antigen processing and presentation, neutrophil chemotaxis, L-serine catabolic process and defence response (Figure 7A). In the molecular function (MF) category, enriched terms included oxygen binding, oxygen transporter activity, heme binding, chemokine activity and L-threonine ammonia-lyase activity (Figure 7B). For the cellular component (CCs), DET-associated genes were primarily localized to the hemoglobin complex, MHC class II protein complex, transcription factor TFIIA complex and haptoglobin–hemoglobin complex (Figure 7C).

KEGG pathway enrichment analysis demonstrated that DET-associated genes were predominantly enriched in cytokine–cytokine receptor interaction, rheumatoid arthritis, cell adhesion molecules, hematopoietic cell lineage, and graft-versus-host disease (Figure 7D).

These results indicate that transcriptomic differences between high- and low-SCC groups are underpinned by genes involved in immune response, antigen presentation, chemotaxis and cytokine signalling, further supporting the central role of immune-related pathways in mastitis susceptibility.

### 3.6. Alternative Splicing Analysis

**Figure 8:**
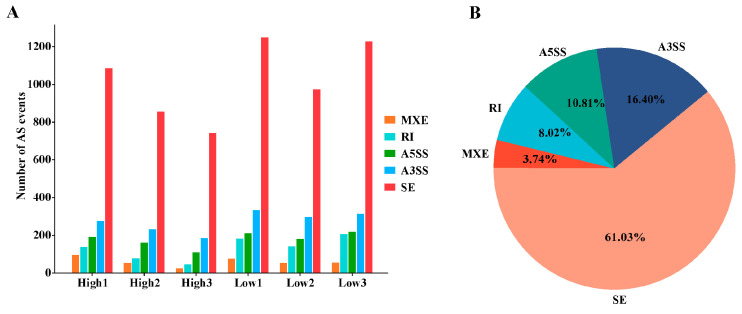
Landscape of AS events across samples. (**A**) Stacked bar plot showing the distribution of AS events across the six individual samples; (**B**) pie chart depicting the proportional distribution of AS event types aggregated across all samples.

To investigate splicing landscape alterations associated with SCC, AS events were identified and quantified in each sample using Astalavista software. Across all six samples, SE represented the most abundant type of AS event, followed by A3SSs and A5SSs (Figure 8A). Notably, the low-SCC group exhibited a higher number of AS events across all categories compared with the high-SCC group, suggesting an overall decrease in AS event frequency with increasing SCC (Figure 8A).

Aggregated analysis of AS events across all samples revealed that SE constituted the majority, accounting for 61.03% of all identified events, followed by A3SSs (16.40%) and A5SSs (10.81%) (Figure 8B).

Differential AS analysis identified 17 events that met the significance threshold (|ΔPSI| > 0.1, *p* < 0.01). These differential AS events were distributed across 15 genes located on 12 chromosomes (Table 2). Chromosome 5 harboured the highest number of differential events, with *MOB3A* and *C5H12orf75* each exhibiting two differential AS events, while the remaining genes each contained a single differential event.

### 3.7. Identification of CXCL2 as a Key Candidate Gene for Mastitis Resistance

To integrate findings from multiple sequencing platforms and prioritize candidate genes for mastitis resistance, we compared the transcriptomic profiles obtained from short-read sequencing (DNBSEQ platform) and long-read sequencing (Oxford Nanopore Technology) in Xinjiang Brown cattle. Previous analysis of short-read data had identified 332 DEGs and 389 DSGs between high- and low-SCC groups [15]. In the present study, long-read sequencing yielded 226 DEGs and 15 DSGs from the same comparison.

**Figure 9:**
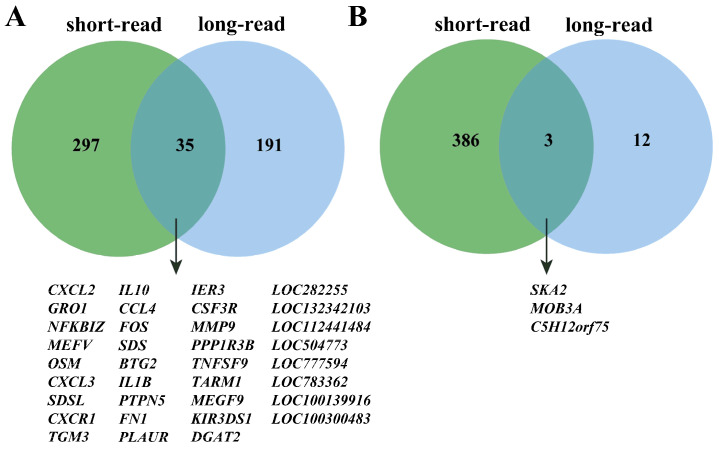
Integration of short-read and long-read sequencing datasets for candidate gene identification. (**A**) Venn diagram showing the overlap of DEGs identified by short-read sequencing and long-read sequencing between high- and low-SCC groups; (**B**) Venn diagram illustrating the overlap of DSGs identified by short-read sequencing and long-read sequencing.

Venn diagram analysis revealed an overlap of 35 DEGs and 3 DSGs between the two platforms, which were considered high-confidence candidates for mastitis resistance (Figure 9). Among the 35 overlapping DEGs, particular attention was given to those with established roles in mastitis pathogenesis, including members of the chemokine and interleukin families. Based on the integration of differential expression levels, pathway enrichment patterns, and functional relevance, *CXCL2* emerged as a top candidate gene for mastitis resistance in Xinjiang Brown cattle.

**Figure 10:**
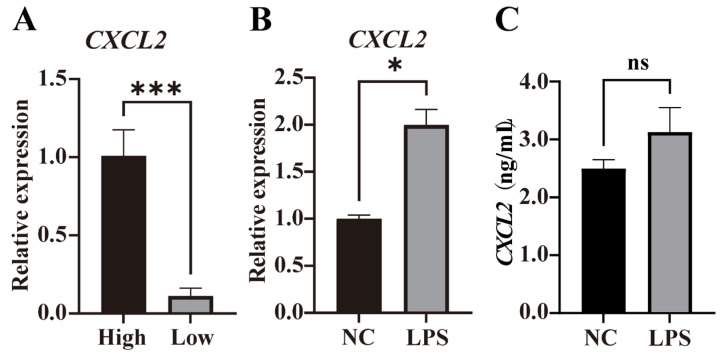
Validation of *CXCL2* expression at transcript and protein levels. (**A**) Relative expression of *CXCL2* mRNA in peripheral blood of Xinjiang Brown cattle from high- and low-SCC groups; (**B**) relative expression of *CXCL2* mRNA in Mac-T following LPS stimulation (3 h); (**C**) concentration of CXCL2 protein in Mac-T culture supernatant following LPS stimulation. Statistical significance was determined by two-tailed Student’s *t*-test. * *p* < 0.05, *** *p* < 0.001; ns indicates not significant (*p* ≥ 0.05).

To validate the expression pattern of the candidate gene *CXCL2* identified through transcriptomic analysis, we examined its transcript and protein levels in both in vivo and in vitro experimental systems using RT-qPCR and enzyme-linked immunosorbent assay (ELISA). Consistent with the sequencing results, *CXCL2* transcript levels were significantly higher in the peripheral blood of Xinjiang Brown cattle from the high-SCC group compared with the low-SCC group (*p* < 0.01; Figure 10A), confirming the upregulation of this gene under conditions of elevated SCC. To further investigate the inflammatory response of *CXCL2*, we established an in vitro inflammation model by stimulating Mac-T bovine mammary epithelial cells with LPS. Following LPS treatment, *CXCL2* expression was significantly upregulated compared with untreated control cells (*p* < 0.05; Figure 10B), indicating that *CXCL2* responds transcriptionally to inflammatory stimuli. To determine whether increased transcript levels translated to elevated protein production, we measured CXCL2 concentrations in cell culture supernatants by ELISA. Consistent with the mRNA expression data, LPS treatment resulted in higher CXCL2 protein levels compared with control conditions (Figure 10C), confirming that *CXCL2* upregulation occurs at both transcriptional and translational levels in response to inflammation.

Collectively, these results demonstrate that *CXCL2* expression is consistently elevated under inflammatory conditions—both in vivo in cattle with high-SCCs and in vitro in LPS-stimulated mammary epithelial cells—supporting its potential role as a molecular marker for mastitis susceptibility and inflammatory response in dairy cattle.

## 4. Discussion

Bovine mastitis imposes substantial economic burdens on the dairy industry worldwide, primarily through reduced milk yield, increased culling rates, and associated management costs [16]. To date, extensive research has elucidated the pathogenic mechanisms, preventive strategies, and predictive models for mastitis, predominantly in Holstein cattle [17]. However, compared with the Holstein breed, investigations into mastitis susceptibility and resistance in Xinjiang Brown cattle—a locally adapted dual-purpose breed in China—remain in their early stages. Elucidating the molecular basis of mastitis resistance in this understudied breed is therefore of considerable scientific and practical importance.

In this study, we employed Oxford Nanopore full-length transcriptome sequencing to profile six Xinjiang Brown cattle, comprising three individuals with high-SCC and three with low-SCC. PCA revealed that samples from the low-SCC group clustered tightly together, indicating good reproducibility among healthy individuals. By contrast, although the high-SCC samples were clearly separable from the low-SCC group, they exhibited greater dispersion within the group. This variability may reflect heterogeneity in the underlying pathogenic pathways or the specific mastitis-causing pathogens affecting individual animals. To date, more than 150 bacterial species have been associated with bovine mastitis [18], including *Staphylococcus aureus* as a major contagious pathogen [19] and various other species [20]. The observed transcriptional variability within the high-SCC group may therefore reflect differential host responses to distinct microbial challenges, underscoring the complexity of mammary gland immune regulation under infection conditions.

Comparative transcriptomic analysis between Xinjiang Brown cattle with high- and low-SCC identified 226 DEGs and 441 DETs. Hierarchical clustering of DEGs and DETs revealed two distinct expression modules: Group-1, characterized by elevated expression in the low-SCC group, and Group-2, comprising genes and transcripts upregulated in the high-SCC group. GO enrichment analysis of Group-2 genes revealed significant associations with immune response, chemokine signalling pathways, antimicrobial humoral immune responses mediated by antimicrobial peptides, and inflammatory responses. These functional annotations establish a direct link between the transcriptional profile of the high-SCC group and mastitis-related biological processes. The enrichment of immune-related pathways in Group-2 indicates that elevated SCC reflects an activated mammary immune state, consistent with the host response to intramammary infection.

To identify key regulators of mastitis resistance in Xinjiang Brown cattle, we performed PPI network analysis using the Degree algorithm. This approach identified several hub genes—including *CXCL2*, *IL1B*, *IL10* and *GRO1*—that were also recognized as high-priority candidates in our previous short-read sequencing dataset, demonstrating cross-platform consistency in the transcriptomic signatures associated with SCC. Among these hub genes, the cytokines *IL1B* and *IL10* exhibited marked upregulation under inflammatory conditions [5], and *GRO1* has been shown to be significantly induced in mammary epithelial cells following inflammatory challenge [21]. *CCL4* (also known as *MIP-1β*), a specific ligand for *CCR5* and member of the macrophage inflammatory protein family, plays a critical role in recruiting pro-inflammatory cells to sites of injury or infection, thereby coordinating both acute and chronic inflammatory host responses [22]. Within the chemokine family, *CXCR1* (the receptor for *IL-8*) has been extensively studied in the context of mastitis susceptibility; single-nucleotide polymorphisms in *CXCR1* are associated with SCS [23] and influence mastitis incidence in dairy cattle [24,25]. *PTPRC* (also known as *CD45*) participates in cytokine signalling and modulates multiple receptors, thereby influencing the production and release of cytokines and regulating inflammatory responses. In addition, *PTPRC* indirectly modulates inflammation by affecting the activation status and function of immune cells such as T cells and B cells [26]. Collectively, these findings reinforce the central role of immune-related genes—particularly those involved in chemokine signalling and cytokine regulation—in determining mastitis resistance in Xinjiang Brown cattle and provide a panel of candidate markers for further functional validation and genetic improvement programmes.

AS analysis revealed a striking discrepancy between sequencing platforms: only 15 DSGs were identified using ONT full-length transcriptome sequencing, whereas short-read sequencing yielded 389 DSGs—approximately 26-fold more. Although short-read sequencing remains widely used, its inherent limitation in capturing full-length RNA sequences can lead to fragmented transcript assembly and erroneous isoform annotation [27]. By contrast, long-read transcriptome technologies overcome the constraints of short read lengths, circumvent amplification biases and capture complete, full-length transcript isoforms [11]. The substantially lower number of DSGs detected by ONT in this study may therefore reflect the higher sensitivity and accuracy of long-read sequencing in resolving genuine splicing events, effectively filtering out artefacts introduced by short-read assembly. The three DSGs commonly identified by both platforms—*C5H12orf75*, *SKA2* and *MOB3A*—have been primarily associated with regulatory functions in tumorigenesis and other diseases. *C5H12orf75* (also known as *OCC-1*) was initially reported as an upregulated gene in colon cancer; its three alternatively spliced isoforms differentially regulate Wnt signalling [28]. In breast cancer, all four isoforms of *C5H12orf75* show elevated expression compared with non-tumour tissues, and increased expression of this gene is correlated with tumorigenesis [29]. *SKA2*, located on chromosome 19, encodes a protein involved in regulating the biological functions of tumour cells. It forms a gene pair with *PRR11* that modulates tumour progression, and altered *SKA2* expression accompanies oncogenesis. Elevated *SKA2* expression has been documented in multiple cancer types, including lung [30], breast [31] and oesophageal [32] cancers. *MOB3A* has been implicated in the pathogenesis of Alzheimer’s disease [33], suggesting broader roles for these shared DSGs beyond mammary gland physiology.

Integration of DEGs and DSGs identified from second-generation (short-read) and Oxford Nanopore full-length transcriptome sequencing yielded 35 high-priority DEGs. Among these, members of the chemokine family—including *CXCL2*, *GRO1*, *CXCL3*, *CCL4* and *CXCR1*—and cytokine genes such as *IL10* and *IL1B* were prominently represented. This enrichment is biologically compelling. During mammary gland infection, chemokines and cytokines are rapidly released to mediate the recruitment of leukocytes to sites of microbial invasion [34]. Consequently, these molecular families are intimately involved in orchestrating inflammatory responses and immune defence mechanisms and are likely to play critical roles in the initiation and progression of mastitis. Chemokines constitute a specialized class of cytokines that direct the targeted migration of leukocytes. They are fundamental to immune system development, inflammatory response, and both innate and adaptive immunity. Owing to their pivotal role in recruiting leukocytes during inflammation, chemokines are frequently classified as inflammatory mediators [35]. Based on integration of differential expression levels and pathway enrichment patterns, we focused on *CXCL2* as a potential molecular marker for mastitis resistance. *CXCL2* is a key member of the CXC chemokine family and exerts its biological functions primarily through binding to its G protein-coupled receptor, *CXCR2*. The CXCL2–CXCR2 axis plays a central role in orchestrating inflammatory responses. Beyond promoting the chemotaxis and recruitment of neutrophils and other immune cells to infection sites, this signalling axis activates multiple downstream pathways—including NF-κB and MAPK cascades—thereby regulating the production of inflammatory mediators and influencing diverse cellular processes such as proliferation, differentiation, apoptosis, migration and adhesion [36]. These multifaceted functions position *CXCL2* as a compelling candidate for modulating mastitis susceptibility and shaping the host response to intramammary infection in dairy cattle.

Mammary epithelial cells constitute the first line of defence against invading pathogens, functioning as a physical barrier while also playing critical roles in immune recognition and response during intramammary infection. The immune system of the mammary gland comprises both innate and adaptive arms, which cooperate to maintain tissue homeostasis and preserve normal lactation function [37,38]. Infection of mammary tissue with Escherichia coli frequently triggers acute mastitis in dairy cattle. Lipopolysaccharide (LPS), the primary toxic component of the *E. coli* cell wall, plays a pivotal role in the initiation and progression of inflammatory responses. Prolonged exposure of cells to LPS stimulation induces a robust inflammatory reaction; consequently, LPS challenge is widely employed to establish in vitro models of mastitis for investigating inflammatory responses [38]. In the present study, we established an LPS-induced inflammatory model in bovine mammary epithelial cells (Mac-T) to examine *CXCL2* expression under inflammatory versus normal conditions. Consistent with our in vivo findings in high-SCC cattle, *CXCL2* transcript levels were significantly upregulated following LPS stimulation. These results suggest that *CXCL2* may serve as a potential molecular indicator of inflammatory status in the mammary gland.

LPS, as the major cell wall component of Gram-negative bacteria, is recognized by Toll-like receptors (TLRs) expressed on bovine mammary epithelial cells. Ligand binding activates multiple intracellular signalling cascades—including calcium signalling pathways and NF-κB—thereby promoting the synthesis and release of pro-inflammatory cytokines and chemokines. These mediators direct the migration of immune cells to sites of infection and amplify the inflammatory response, leading to physiological changes in mammary epithelial cells such as cellular swelling and increased permeability [39]. The observed upregulation of *CXCL2* following LPS treatment in our study is consistent with this established mechanism. Notably, lipoteichoic acid (LTA), a major cell wall component of Gram-positive bacteria, is also used to establish inflammatory models of mastitis. Studies have demonstrated that LTA induces inflammatory responses in mammary tissue in vivo and significantly promotes the release of chemokines—including *CXCL1*, *CXCL2* and *CXCL3*—thereby mediating the recruitment of neutrophils and other immune cells [40]. These findings indicate that, regardless of whether inflammation is triggered by Gram-negative (LPS) or Gram-positive (LTA) bacterial components, chemokines such as *CXCL2* are consistently upregulated and actively participate in regulating the inflammatory response. Collectively, our results from both in vivo and in vitro models demonstrate that *CXCL2* expression is robustly induced under inflammatory conditions, supporting its potential utility as a molecular marker for mastitis susceptibility and as a candidate target for genetic improvement programmes aimed at enhancing mammary health in dairy cattle. These findings suggest that *CXCL2* could be incorporated into marker-assisted or genomic selection strategies for improving mastitis resistance in Xinjiang Brown cattle. Future studies should focus on validating its predictive power across breeds and assessing possible pleiotropic effects.

This study systematically constructed a full-length peripheral blood transcriptome atlas of Xinjiang Brown cattle with varying somatic cell counts and identified CXCL2 as a potential molecular marker for mastitis resistance. However, several limitations should be acknowledged. First, although animals in the high-SCC group exceeded the threshold commonly used for clinical mastitis, clinical symptoms were not systematically recorded. Thus, it was not possible to definitively distinguish between clinical and subclinical mastitis. Second, the sample size was relatively small (*n* = 3 per group, total *n* = 6), which may limit statistical power. Third, the study was restricted to Xinjiang Brown cattle; therefore, the generalizability of the findings, especially regarding *CXCL2*, to other breeds requires further validation.

## 5. Conclusions

In this study, we employed ONT full-length transcriptome sequencing to systematically delineate gene expression profiles in peripheral blood leukocytes of Xinjiang Brown cattle stratified by SCCs. Comparative analysis between high- and low-SCC groups revealed that the high-SCC group exhibited significant activation of immune response, inflammatory response, neutrophil chemotaxis, and chemokine-related pathways, with *CXCL2*, *IL1B* and *IL10* identified as core differentially expressed genes. Integration of short-read sequencing data with our ONT dataset further prioritized *CXCL2* as a key candidate gene associated with mastitis susceptibility. Validation by RT–qPCR and ELISA confirmed that *CXCL2* expression was significantly upregulated in both the peripheral blood of high-SCC individuals and in an LPS-induced inflammatory model of Mac-T bovine mammary epithelial cells. Consistent with the transcript-level findings, CXCL2 protein concentrations were significantly elevated in cell culture supernatants following LPS stimulation. Collectively, these findings establish *CXCL2* as a promising molecular marker for mastitis resistance breeding in Xinjiang Brown cattle. This study provides a foundational resource for elucidating the molecular mechanisms underlying mammary gland health and offers potential targets for precision selection programmes aimed at improving disease resistance and production performance in dairy cattle.

## Figures and Tables

**Table 1:** RT qPCR primer sequence.

Gene	Primer Sequence (5′ to 3′)	Product Size (bp)
*CXCL2*	F:ATATTTCTGAGGAGCCCACATTATGCC	84
	R:CCACCCGACAATGAGTTCACTATCTG	
*GAPDH*	F:CACTGAGGACCAGGTTGTCT	119
	R:TGTCGTACCAGGAAATGAGC	

**Table 2:** Differential splicing events and genes.

Gene	Type of Alternative Splicing	Chromosome	ΔPSI	*p* Value
*MOB3A*	SSE	7	17.55	0.0052
*MOB3A*	MSE	7	13.88	0.0021
*TAF1D*	A3SS	29	14.74	0.0023
*TRIM34*	SSE	15	12.2	0.0020
*LOC104968422*	SSE	18	12.02	0.0073
*MAPRE1*	A5SS	13	10.01	0.0035
*LOC507055*	SSE	3	−10.15	0.0024
*FBXO6*	A3SS	16	−10.17	0.0043
*RNF121*	SSE	15	−10.41	0.0095
*PLGRKT*	MSE	8	−10.95	0.0071
*SKA2*	MSE	19	−11.82	0.0034
*SLC25A17*	MSE	5	−13.75	3.96 × 10^−6^
*ABHD16A*	MSE	23	−16.3	7.9 × 10^−4^
*C5H12orf75*	SSE	5	−20.03	4.5 × 10^−3^
*C5H12orf75*	SSE	5	−31.49	9.4 × 10^−3^
*ONT.17045*	SSE	23	17.43	8.6 × 10^−3^
*ONT.10887*	SSE	14	−25.46	8.8 × 10^−3^

Note: SSE represents single exon skipping, while MSE represents multiple exons skipping.

## Data Availability

The data that support the findings of this study are available from the corresponding author, X.H., upon reasonable request.

## Supplementary Materials

The following supporting information can be downloaded at: https://www.mdpi.com/article/10.3390/biology15110830/s1, Figure S1: Statistics on the distribution of reads length; Table S1: RNA concentration and quality detection; Table S2: Statistics of full-length transcriptome sequencing data of Xinjiang Brown cattle; Table S3: Statistics of full-length sequence data; Table S4: Comparison results of reference genomes.

## References

[1] Rajala-Schultz P.J., Gröhn Y.T., McCulloch C.E., Guard C.L. (1999). Effects of Clinical Mastitis on Milk Yield in Dairy Cows. J. Dairy Sci..

[2] Martin P., Barkema H.W., Brito L.F., Narayana S.G., Miglior F. (2018). Symposium Review: Novel Strategies to Genetically Improve Mastitis Resistance in Dairy Cattle. J. Dairy Sci..

[3] Schukken Y.H., Wilson D.J., Welcome F., Garrison-Tikofsky L., Gonzalez R.N. (2003). Monitoring Udder Health and Milk Quality Using Somatic Cell Counts. Vet. Res..

[4] Ruegg P.L. (2017). A 100-Year Review: Mastitis Detection, Management, and Prevention. J. Dairy Sci..

[5] Wang D., Yang H., Ma S., Liu T., Yan M., Dong M., Zhang M., Zhang T., Zhang X., Xu L. (2024). Transcriptomic Changes and Regulatory Networks Associated with Resistance to Mastitis in Xinjiang Brown Cattle. Genes.

[6] Sharun K., Dhama K., Tiwari R., Gugjoo M.B., Iqbal Yatoo M., Patel S.K., Pathak M., Karthik K., Khurana S.K., Singh R. (2021). Advances in Therapeutic and Managemental Approaches of Bovine Mastitis: A Comprehensive Review. Vet. Q..

[7] Cheng Z., Buggiotti L., Salavati M., Marchitelli C., Palma-Vera S., Wylie A., Takeda H., Tang L., Crowe M.A., Wathes D.C. (2021). Global Transcriptomic Profiles of Circulating Leucocytes in Early Lactation Cows with Clinical or Subclinical Mastitis. Mol. Biol. Rep..

[8] Zhou J., Liu L., Chen C.J., Zhang M., Lu X., Zhang Z., Huang X., Shi Y. (2019). Genome-Wide Association Study of Milk and Reproductive Traits in Dual-Purpose Xinjiang Brown Cattle. BMC Genom..

[9] Zhong L., Ma S., Wang D., Zhang M., Tian Y., He J., Zhang X., Xu L., Wu C., Dong M. (2023). Methylation Levels in the Promoter Region of FHIT and PIAS1 Genes Associated with Mastitis Resistance in Xinjiang Brown Cattle. Genes.

[10] Wang D., Ma S., Yan M., Dong M., Zhang M., Zhang T., Zhang T., Zhang X., Xu L., Huang X. (2024). DNA Methylation Patterns in the Peripheral Blood of Xinjiang Brown Cattle with Variable Somatic Cell Counts. Front. Genet..

[11] Maitra R.D., Kim J., Dunbar W.B. (2012). Recent Advances in Nanopore Sequencing. Electrophoresis.

[12] Chen Z., Xie L., Tang X., Zhang Z. (2023). Recombination Map Construction Method Using ONT Sequence. MethodsX.

[13] Li H. (2018). Minimap2: Pairwise Alignment for Nucleotide Sequences. Bioinformatics.

[14] Foissac S., Sammeth M. (2007). ASTALAVISTA: Dynamic and Flexible Analysis of Alternative Splicing Events in Custom Gene Datasets. Nucleic Acids Res..

[15] Yan M.J., Wang D., Ma S.C., Zhang T., Zhang M.H., Huang X.X. (2025). Analysis of alternative splicing events and differentially expressed genes in Xinjiang Brown Cattle with high and low somatic cell counts. Chin. J. Anim. Sci..

[16] Corrêa D.C., Nunes G.T., Barcelos R.A.D., Dos Santos J.R., Vogel F.S.F., Cargnelutti J.F. (2024). Economic Losses Caused by Mastitis and the Influence of Climate Variation on the Occurrence of the Disease in a Dairy Cattle Farm in Southern Brazil. Trop. Anim. Health Prod..

[17] Satoła A., Satoła K. (2024). Performance Comparison of Machine Learning Models Used for Predicting Subclinical Mastitis in Dairy Cows: Bagging, Boosting, Stacking and Super-Learner Ensembles versus Single Machine Learning Models. J. Dairy Sci..

[18] Shome B.R., Das Mitra S., Bhuvana M., Krithiga N., Velu D., Shome R., Isloor S., Barbuddhe S.B., Rahman H. (2011). Multiplex PCR Assay for Species Identification of Bovine Mastitis Pathogens: PCR for Detection of Mastitis Pathogens. J. Appl. Microbiol..

[19] Saeed S.I., Kamaruzzaman N.F., Gahamanyi N., Nguyen T.T.H., Hossain D., Kahwa I. (2024). Confronting the Complexities of Antimicrobial Management for Staphyloccous Aureus Causing Bovine Mastitis: An Innovative Paradigm. Ir. Vet. J..

[20] Shrinet G., Chhabra R., Sharma A., Batra K., Talukdar S.J., Maan S. (2023). High Throughput Luminex Beads Based Multiplex Assay for Identification of Six Major Bacterial Pathogens of Mastitis in Dairy Animals. Front. Cell. Infect. Microbiol..

[21] Liu J., Gao Y., Zhang X., Hao Z., Zhang H., Gui R., Liu F., Tong C., Wang X. (2024). Transcriptome Sequencing Analysis of Bovine Mammary Epithelial Cells Induced by Lipopolysaccharide. Anim. Biotechnol..

[22] Maurer M., Von Stebut E. (2004). Macrophage Inflammatory Protein-1. Int. J. Biochem. Cell Biol..

[23] Leyva-Baca I., Schenkel F., Martin J., Karrow N.A. (2008). Polymorphisms in the 5′ Upstream Region of the CXCR1 Chemokine Receptor Gene, and Their Association with Somatic Cell Score in Holstein Cattle in Canada. J. Dairy Sci..

[24] Pawlik A., Sender G., Kapera M., Korwin-Kossakowska A. (2015). Experimental Immunology Association between Interleukin 8 Receptor α Gene (CXCR1) and Mastitis in Dairy Cattle. Cent. Eur. J. Immunol..

[25] Pokorska J., Dusza M., Kułaj D., Żukowski K., Makulska J. (2016). Single Nucleotide Polymorphisms in the CXCR1 Gene and Its Association with Clinical Mastitis Incidence in Polish Holstein-Friesian Cows. Genet. Mol. Res..

[26] Al Barashdi M.A., Ali A., McMullin M.F., Mills K. (2021). Protein Tyrosine Phosphatase Receptor Type C (PTPRC or CD45). J. Clin. Pathol..

[27] Wang B., Tseng E., Regulski M., Clark T.A., Hon T., Jiao Y., Lu Z., Olson A., Stein J.C., Ware D. (2016). Unveiling the Complexity of the Maize Transcriptome by Single-Molecule Long-Read Sequencing. Nat. Commun..

[28] Najafi H., Soltani B.M., Dokanehiifard S., Nasiri S., Mowla S.J. (2017). Alternative Splicing of the OCC-1 Gene Generates Three Splice Variants and a Novel Exonic microRNA, Which Regulate the Wnt Signaling Pathway. RNA.

[29] Ghalaei A., Kay M., Zarrinfam S., Hoseinpour P., Behmanesh M., Soltani B.M. (2018). Overexpressed in Colorectal Carcinoma Gene (OCC-1) Upregulation and APPL2 Gene Downregulation in Breast Cancer Specimens. Mol. Biol. Rep..

[30] Wang Y. (2015). The Gene Pair PRR11 and SKA2 Shares a NF-Y-Regulated Bidirectional Promoter and Contributes to Lung Cancer Development. Biochim. Biophys. Acta (BBA)-Gene Regul. Mech..

[31] Wang Y., Zhang C., Mai L., Niu Y., Wang Y., Bu Y. (2019). PRR11 and SKA2 Gene Pair Is Overexpressed and Regulated by P53 in Breast Cancer. BMB Rep..

[32] Chen J., Yang H.-M., Zhou H.-C., Peng R.-R., Niu Z.-X., Kang C.-Y. (2020). PRR11 and SKA2 Promote the Proliferation, Migration and Invasion of Esophageal Carcinoma Cells. Oncol. Lett..

[33] Huang Z., Wang H., Wang D., Zhao X., Liu W., Zhong X., He D., Mu B., Lu M. (2022). Identification of Core Genes in Prefrontal Cortex and Hippocampus of Alzheimer’s Disease Based on mRNA-miRNA Network. J. Cell. Mol. Med..

[34] Satheesan L., Dang A.K., Alex R. (2025). Cytokine Interactions and Chemokine Dysregulations in Mastitis Immunopathogenesis: Insights from Transcriptomic Profiling of Milk Somatic Cells in Tropical Sahiwal (Bos Indicus) Cows. Front. Immunol..

[35] Zlotnik A., Yoshie O. (2012). The Chemokine Superfamily Revisited. Immunity.

[36] Lv Y., Chen C., Han M., Tian C., Song F., Feng S., Xu M., Zhao Z., Zhou H., Su W. (2025). CXCL2: A Key Player in the Tumor Microenvironment and Inflammatory Diseases. Cancer Cell Int..

[37] Rainard P., Riollet C. (2006). Innate Immunity of the Bovine Mammary Gland. Vet. Res..

[38] Wellnitz O., Bruckmaier R.M. (2012). The Innate Immune Response of the Bovine Mammary Gland to Bacterial Infection. Vet. J..

[39] Islam M.A., Takagi M., Fukuyama K., Komatsu R., Albarracin L., Nochi T., Suda Y., Ikeda-Ohtsubo W., Rutten V., Eden W.V. (2020). Transcriptome Analysis of The Inflammatory Responses of Bovine Mammary Epithelial Cells: Exploring Immunomodulatory Target Genes for Bovine Mastitis. Pathogens.

[40] Rainard P., Fromageau A., Cunha P., Gilbert F.B. (2008). Staphylococcus Aureus Lipoteichoic Acid Triggers Inflammation in the Lactating Bovine Mammary Gland. Vet. Res..

