# Supplementary material for: Differences in Peripheral Blood Gene Expression of Xinjiang Brown Cattle with Varying Somatic Cell Counts
Source: Biology (Basel). 2026 May 25;15(11):830. doi: 10.3390/biology15110830 (PMC13255933; doi:10.3390/biology15110830)
Supplement: Supplementary file 1 [file biology-15-00830-s001.zip › biology-4310003-supplementary.pdf]

Table S1　RNA concentration and quality detection

Sample	Concentration（ng/μL）	Total（μg）	RIN	OD260/280
High1	156.2	4.69	7.8	1.86
High2	139.7	4.19	8.1	1.83
High3	108.9	3.27	6.5	1.82
Low1	226.3	6.79	7.5	1.95
Low2	203.0	6.09	8.0	1.82
Low3	261.9	7.86	8.0	1.93

Table S2　Statistics of full-length transcriptome sequencing data of Xinjiang Brown cattle

Sample	Raw reads	Clean reads	Raw bases	N50 length	Mean length	Max length
High1	8 044 416	7 177 142	6 415 673 097	787	797	144 107
High2	8 421 404	7 546 917	6 473 152 312	773	768	190 233
High3	9 389 146	8 447 986	6 561 460 394	713	698	117 028
Low1	6 996 830	5 475 643	5 955 526 441	893	851	133 494
Low2	8 774 006	7 833 701	6 953 910 568	804	792	132 321
Low3	8 115 348	7 225 492	6 607 287 288	814	814	101 652

Table S3　Statistics of full-length sequence data

Sample	Clean reads	Full-length reads	Full-length rate
High1	7 704 506	7 177 142	93.16%
High2	8 052 910	7 546 917	93.72%
High3	8 943 602	8 447 986	94.46%
Low1	5 851 265	5 475 643	93.58%
Low2	8 434 944	7 833 701	92.87%
Low3	7 771 061	7 225 492	92.98%

Table S4　Comparison results of reference genomes

Sample	Total Reads	Mapped reads	Mapped rate
High1	7 177 142	7 057 026	98.33%
High2	7 546 917	7 401 736	98.08%
High3	8 447 986	8 148 910	96.46%
Low1	5 475 643	5 247 844	95.84%
Low2	7 833 701	7 632 881	97.44%
Low3	7 225 492	7 072 155	97.88%

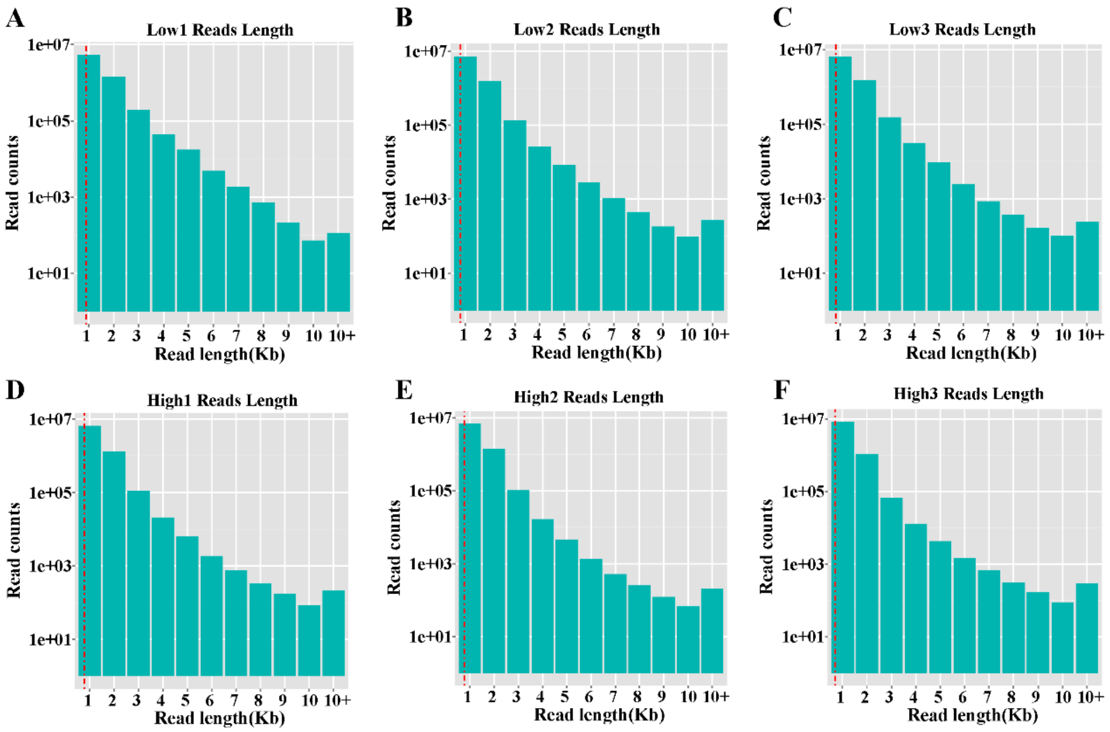

Figure S1 Statistics on the distribution of reads length
